# Supplementary material for: Are reads required? High-precision variant calling from bacterial genome assemblies
Source: Access Microbiol. 2025 May 28;7(5):001025.v3. doi: 10.1099/acmi.0.001025.v3 (PMC12120141; doi:10.1099/acmi.0.001025.v3)
Supplement: Uncited Supplementary Material 1. [file acmi-7-01025-s001.pdf]

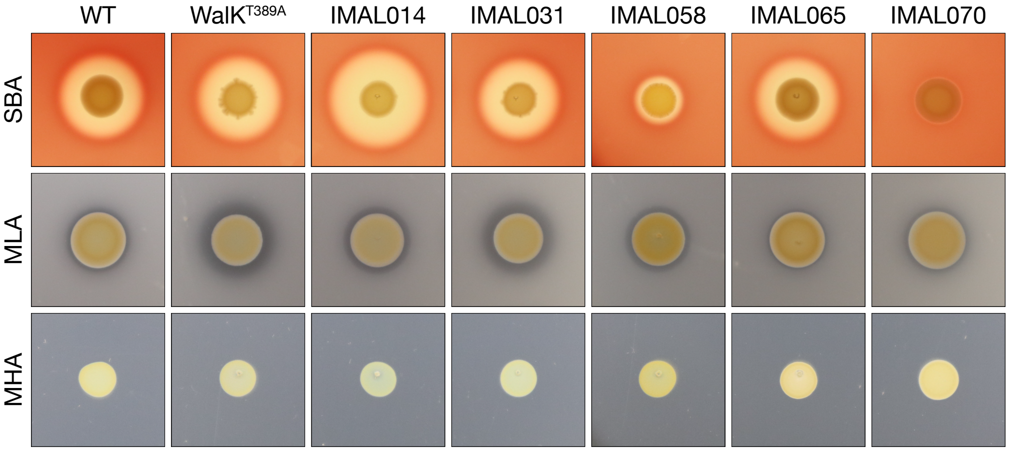

Figure S1: Phenotypic characterisation of the isolates.

SBA = Sheep blood agar (5% sheep blood in Columbia agar); shows haemolytic activity.

MLA = *Micrococcus luteus* agar (BHI agar containing 0.2% Micrococcus luteus cells [Sigma-Aldrich, M3770]); shows the secretion/activity of one WalR-regulated peptidoglycan hydrolase (Atl).

MHA = Mueller-Hinton agar (Difco); shows the colour of the cells. IMAL058 is a SigB mutant, making it more yellow than the other isolates.

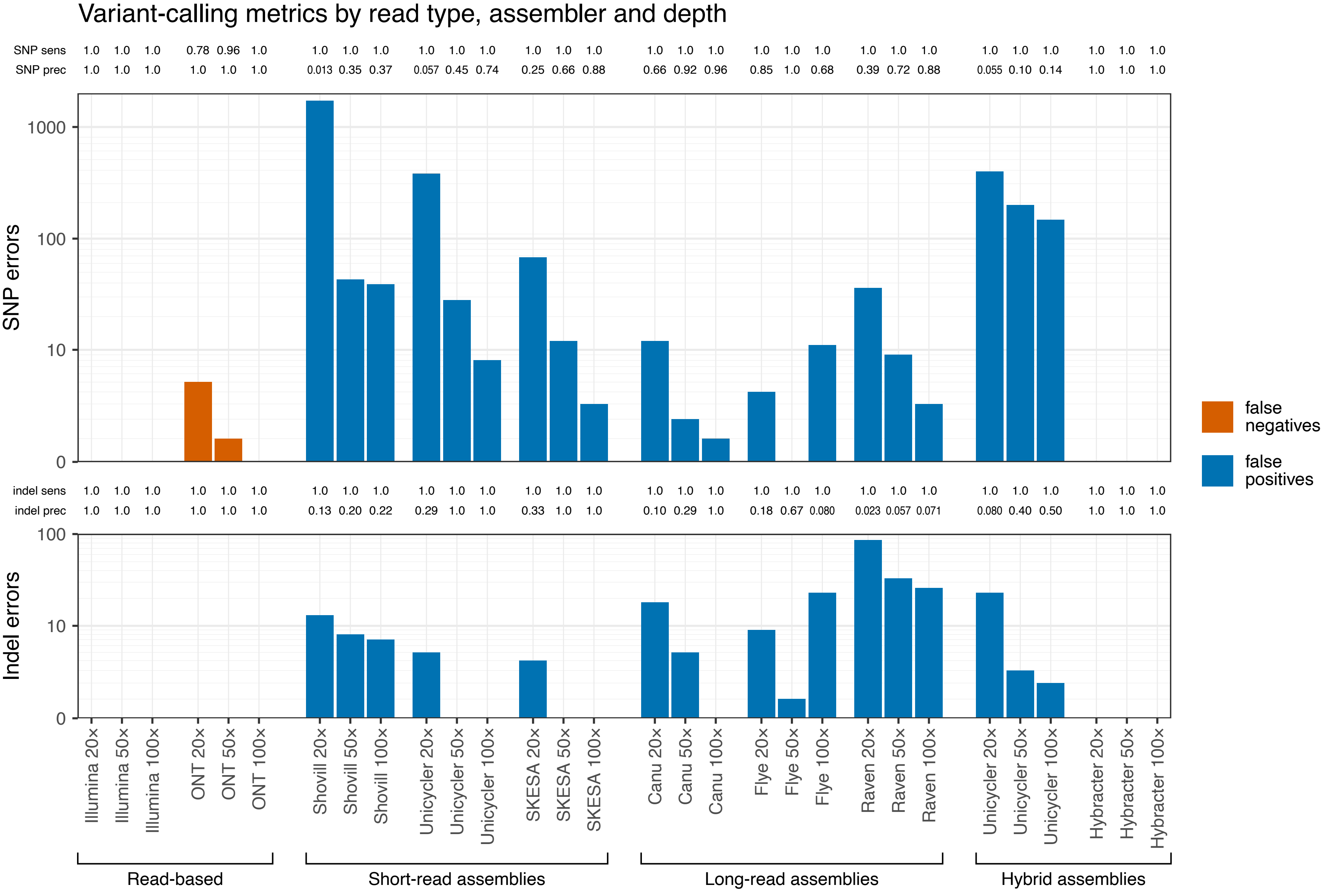

Figure S2: Variant calling metrics for both read- and assembly-based variant calling methods at each read depth. False negative and false positive errors are shown in the plots. Sensitivity (sens) and precision (prec) are shown above the plots. The y-axes have a pseudo-log transformation.

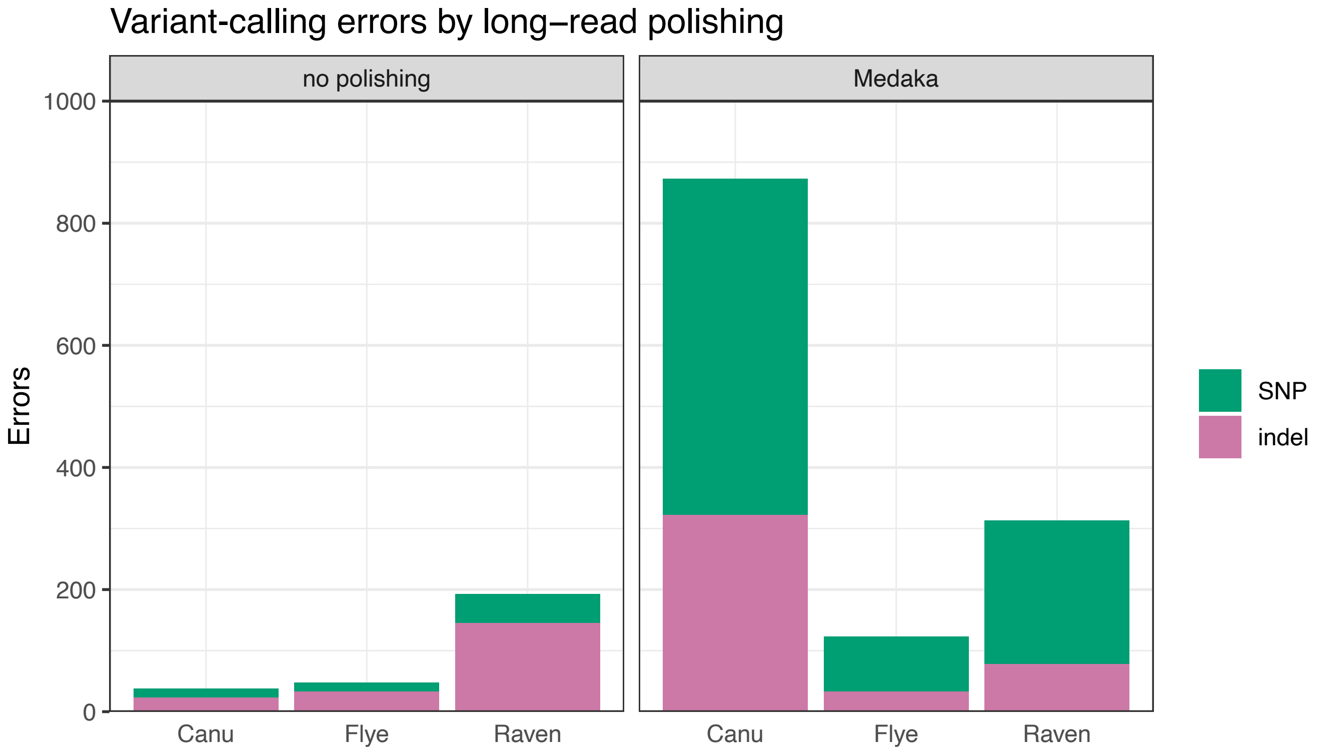

Figure S3: Variant-calling errors, before and after Medaka polishing, for each of the long-read assembly methods.
